# Substance use amongst adult patients admitted to an irish acute mental health unit

**DOI:** 10.1192/j.eurpsy.2021.1510

**Published:** 2021-08-13

**Authors:** A. Duggan, N. Murray, S. Buckley, G. Lalevic

**Affiliations:** Department Of Psychiatry, St. Stephen’s Hospital, Cork, Ireland

**Keywords:** Mental illness, Addiction, Substance use, acute mental health unit

## Abstract

**Introduction:**

Comorbid substance misuse in mental illness presents a significant challenge to mental health services. It may lead to higher rates of relapse, hospital admissions and poorer treatment outcomes. Up to 47% of inpatients in Irish mental health units may experience substance misuse. Despite the Irish government’s ‘Vision for Change’ policy (2006), access to specialised services remains variable.

**Objectives:**

Evaluate: -prevalence of substance misuse at an Irish mental health unit. -quality and detail of the recorded substance misuse history. -access to specialised services for patients experiencing substance misuse.

**Methods:**

A retrospective chart review of inpatients in a mental health unit over 12 months, was completed. Information recorded included: demographic details, diagnosis, substance use history; access to substance misuse services. Microsoft Excel was utilised for data input and analysis.

**Results:**

267 patients were admitted over twelve months. Substance misuse was the primary diagnosis of 6% and the secondary diagnosis of 67%. 46% of patients reported current substance misuse, 52% reported historical substance misuse. Frequency and quantity of use was documented in 65% and 48% of cases respectively. 4% of patients with a substance misuse history were in current contact with addiction services.

**Conclusions:**

Although 46% of patients reported substance misuse, only 4% were in contact with specialised addiction services. This highlights a significant unmet need. There was variability in the quality of the recorded substance misuse history. In order to fully understand comorbid substance misuse, this be addressed. The addition of a more formatted substance misuse section, to admission proformas, may help to alleviate this issue.

